# Impact of extended pre-scan written instructions on motion artifacts during head magnetic resonance imaging

**DOI:** 10.25122/jml-2022-0133

**Published:** 2022-09

**Authors:** Suliman Salih, Osamah Abdulaal, Moawia Gameraddin, Mustafa Alhasan, Mohamed Hasaneen

**Affiliations:** 1 Department of Radiology and Medical Imaging, Fatima College of Health Sciences, Abu Dhabi, United Arab Emirates; 2 Department of Diagnostic Radiologic Technology, Faculty of Applied Medical Sciences, Taibah University, Medina, Saudi Arabia; 3 Radiologic Technology Program, Applied Medical Sciences College, Jordan University of Science and Technology, Irbid, Jordan

**Keywords:** pre-scan written instructions, magnetic resonance imaging (MRI), motion artifacts

## Abstract

This study aimed to assess the effect of extending pre-scan written instructions to patients undergoing head magnetic resonance imaging (MRI) examinations on motion artifacts. A controlled study was conducted in King Fahad Hospital at the Department of Radiology. A total of 100 patients were involved: 50 received only routine oral hospital instructions (control group), and another 50 received pre-scan extended written instructions besides routine oral hospital instructions (intervention group). The head MRI images were assessed regarding motion artifacts. Informed consent was obtained from all patients included in the study. The incidence of motion artifacts was significantly less in the intervention group than in the control group, 10% and 58%, respectively (p-value=0.001). The motion artifacts decreased significantly in the intervention group compared to the control group. Extending written information before an MRI scan could significantly reduce motion artifacts and improve image quality.

## INTRODUCTION

Magnetic resonance imaging (MRI) is a radiological examination method. It is a non-invasive and safe method which does not use ionizing radiation [1, 2]. However, some patients experience discomforts such as claustrophobia or panic associated with physical confinement and loud noises. These problems might cause movement in some patients during MRI examinations. Patient movement during MRI examination may impair image quality because of motion artifacts [3, 4]. Approximately 70% of the images have been reported to show movement artifacts [4]. Furthermore, on average, 2.3% of patients are unable to complete their scan or obtain a diagnosis [1]. Therefore, these factors not only prevent many patients from reaping the benefits of MRI findings but also represent an issue of socioeconomic importance in the healthcare system [5, 6].

Patient anxiety and dissatisfaction have been associated with some medical procedures in general [7]. The research indicates that informed patients experiencing medical events are less anxious and more satisfied with their care than those less knowledgeable [8]. However, these uncomfortable conditions can be reduced by giving the patients clear instructions about the medical procedures and the details of the health care they will receive [9, 10].

Although some published data from Europe and other countries showed that motion artifacts and patient anxiety could be reduced by providing patients with written instructions about MRI procedures [11–14], to the best of our knowledge, there are no published data from Saudi Arabia about the effect of extending pre-scan written instructions on clinical MRI motion artifacts. It has been reported that cultural differences may affect patients' attitudes towards medical care, including diagnosis and treatment of diseases [15]. The present study aimed to assess whether extending pre-scan written instructions decreases motion artifacts throughout head MRI procedures.

## MATERIAL AND METHODS

A controlled study was conducted in the Department of Radiology at King Fahad Hospital in Madina, Kingdom of Saudi Arabia (KSA). The researchers used two MRI scanners; Siemens Magnetom Vision (1.5T). The duration of the study was 4 months. All patients included in the study received a head MRI scan.

A total of 100 adult patients were included in this study. The patients were divided into two groups: 50 patients were randomly assigned to the control group without receiving any written information before the MRI examination other than the regular hospital oral instructions. The remaining 50 patients were randomly assigned to the study (intervention) group. The intervention groups were provided with extended written information about the MRI examinations before the scan, besides the regular hospital instructions. The inclusion criteria items were: (1) ability to speak, read and understand Arabic and (2) physical and mental ability to complete the questionnaire. The following were the exclusion criteria items: (1) presence of diseases that affect movement, such as Parkinson's disease and (2) refusal to participate. The images were observed and assessed for motion artifacts by two qualified specialists: a radiologist and an MRI technologist (experienced in detecting motion artifacts) with more than 3 years of experience. The observer was blinded to the patients' characteristics during the assessment of the images.

### Statistical analysis

The collected data were analyzed using SPSS software (version 16.00). Comparisons of the parameters were interpreted using the Chi-square test. Spearman correlation test was used to find an association between pre-scan written instructions and MRI motion artifacts. Chi-square values<0.05 were considered significant.

## RESULTS

One hundred adult patients (male=56; female=44; age range=18–77 years; mean age=43±14.19 years) were included in the study.

Table 1 summarizes the number and % of motion artifacts in the intervention and control groups. The number of patients who did not show motion artifacts was 45 (90%) and 21 (42%), respectively, whereas the numbers of patients who showed motion artifacts were 5 (10%) and 29 (58%), respectively. The table also illustrates a significant negative correlation between written information and the occurrence of motion artifacts in both the intervention and control groups. (P-value=0.001, R^2^=0.507).

**Table 1:** The correlation between occurrence of motion artifacts in intervention and control groups.

Motion artifact	Control group n=50 & (%)	Intervention group n=50 & (%)	R-square	P-value
Total number (%) of patients with motion artifacts	29/50 (58%)	5/50 (10%)	R^2^=0.507	0.001

## DISCUSSION

Motion artifact appearance is a common problem in MRI examinations. They degrade image quality and possibly interfere with interpretation [7]. Educating patients about MRI procedures is the best method to ensure that a patient stays still to reduce anxiety and motion artifacts. The purpose of this research was to assess whether the extended pre-scan written information has an impact on reducing motion artifacts in head MR images.

The results of this study revealed that the incidence of motion artifacts was significantly decreased in the intervention group compared to the control group, as illustrated in Table 1. A significant positive association was observed between image motion artifacts and patients in the intervention group who were given extended written instructions. This finding agrees with the first study published by Törnqvist et al. [8], which investigated the effect of extended pre-scan written information on MRI motion artifacts. This study reported that extended pre-scan written instructions significantly decreased MRI motion artifacts [8]. Moreover, a similar conclusion was reached by Syed H. Ali et al., who reported that clinical MRI motion degradation could be reduced by using a pre-scan patient information pamphlet [16].

The result of this study, also in line with many other studies, assessed the effect of written information on decreasing anxiety and indirectly reducing motion artifacts [13, 14]. Reducing MRI motion artifacts is essential in improving image quality and providing proper MRI image reporting. Consequently, this leads to adequate diagnosis and treatment [17–19]. From a broad perspective, effective communication and patient education become crucial elements in the quality of healthcare services [20]. Several studies reported that patient education is positively impacted by reducing patient anxiety and allowing the patient to cooperate during diagnostic and treatment procedures [21–24].

The main limitation of the study is the small sample size and small geographical area of the study. This study is the first one in its location. It will open further research paths. A study with a large sample size and different geographical areas is recommended.

## CONCLUSION

In conclusion, this study showed that pre-written instructions could decrease the incidence of motion artifacts by helping patients understand the importance of movement constraints during MRI examinations. Reducing motion artifacts could improve MRI image quality by enabling an adequate diagnosis.

## Personal thanks

The authors would like to express their gratitude to the staff of the Department of Radiology and Medical Imaging, King Fahad for their cooperation and Dr. Sultan Ashabi for revising the manuscript, and Dr. Mosleh Alrdadi for his support.
